# Cytogenetics and Cytogenomics Evaluation in Cancer

**DOI:** 10.3390/ijms20194711

**Published:** 2019-09-23

**Authors:** Ilda Patrícia Ribeiro, Joana Barbosa Melo, Isabel Marques Carreira

**Affiliations:** 1 Cytogenetics and Genomics Laboratory, Faculty of Medicine, University of Coimbra, 3004-531 Coimbra, Portugal; ildaribeiro.patricia@gmail.com (I.P.R.); mmelo@fmed.uc.pt (J.B.M.); 2 iCBR-CIMAGO, Center of Investigation on Environment, Genetics and Oncobiology, Faculty of Medicine, University of Coimbra, 3004-531 Coimbra, Portugal; 3 CNC, IBILI, Group of Aging and Brain Diseases: Advanced Diagnosis and Biomarkers, 3004-531 Coimbra, Portugal

**Keywords:** high-throughput technologies, biomarkers, genomic alterations, driver mutations

## Abstract

The availability of cytogenetics and cytogenomics technologies improved the detection and identification of tumor molecular signatures as well as the understanding of cancer initiation and progression. The use of large-scale and high-throughput cytogenomics technologies has led to a fast identification of several cancer candidate biomarkers associated with diagnosis, prognosis, and therapeutics. The advent of array comparative genomic hybridization and next-generation sequencing technologies has significantly improved the knowledge about cancer biology, underlining driver genes to guide targeted therapy development, drug-resistance prediction, and pharmacogenetics. However, few of these candidate biomarkers have made the transition to the clinic with a clear benefit for the patients. Technological progress helped to demonstrate that cellular heterogeneity plays a significant role in tumor progression and resistance/sensitivity to cancer therapies, representing the major challenge of precision cancer therapy. A paradigm shift has been introduced in cancer genomics with the recent advent of single-cell sequencing, since it presents a lot of applications with a clear benefit to oncological patients, namely, detection of intra-tumoral heterogeneity, mapping clonal evolution, monitoring the development of therapy resistance, and detection of rare tumor cell populations. It seems now evident that no single biomarker could provide the whole information necessary to early detect and predict the behavior and prognosis of tumors. The promise of precision medicine is based on the molecular profiling of tumors being vital the continuous progress of high-throughput technologies and the multidisciplinary efforts to catalogue chromosomal rearrangements and genomic alterations of human cancers and to do a good interpretation of the relation genotype—phenotype.

## 1. Introduction

Cytogenetics and genomics technologies have demonstrated that cancer is a disease of the cell genome, resulting in clonal expansion of cells that have accumulated the most advantageous set of genetic aberrations, such as point mutations, chromosomal rearrangements, DNA-dosage abnormalities, and alteration of microsatellite [1]. Several chromosome rearrangements are specific of a particular disease, such as the translocation between chromosomes 9 and 22 in chronic myeloid leukaemia (known as the ‘Philadelphia’ chromosome), yielding the BCR-ABL fusion protein [2]. Other chromosomal alterations are consequence of a cellular malignant transformation process, such as isochromosomes 8q and 17q in carcinomas and trisomy 8 in acute myeloblastic leukemia [3]. The presence of specific chromosomal and genetic alterations exclusively observed in malignant cells helps in cancer diagnosis and prognosis, allowing also to quantify residual disease. Several different types and sizes of chromosomal abnormalities can be found in human cancers, being the products of these dysregulated genes and cellular pathways specific targets for new drugs [4]. It is also important to note that some molecular alterations, which are vital in the early phase of tumor development, may be lost or masked by subsequent events or no longer be functionally relevant. Others may be neutral or detrimental to the tumor but be present in a cell that developed a sufficiently protumorigenic aberration [1]. The advent of new high-throughput technologies allows to characterize and decode the whole DNA sequence of cancer genomes as well as to compare the genomic alterations not only between normal and tumor genomes but also between different patients and different tumor types [5]. The next-generation sequencing (NGS) analysis shed light on tumor heterogeneity through the sequencing of spatially and temporally separate tumor regions [6]. With the availability of large-scale cytogenomic technologies and with their decreasing cost, human cancer research has benefited of the identification of new mutations and gene fusions, which contribute to the progress of precision medicine and personalized treatment of cancer patients [7]. Therefore, advances in tumors mutational analysis led to development of new molecular target therapies, such as for *BRAF* and *NRAS* mutations, the genetic alterations most frequently detected in cutaneous melanoma [8]. Additionally, NGS technology allowed the detection of multiple mutations in different genes through the detection and analysis of circulating tumor cells (CTCs) and circulating tumor DNA (ctDNA), which provide a minimally or non-invasive method for early cancer diagnosis and for monitoring the genomic events that occur during cancer progression as well as during and after cancer treatment [9]. In this review, we discuss the usefulness of cytogenetics and cytogenomics analysis in human cancer in order to identify biomarkers with clinical applicability, which contribute to improving the precision of medicine in oncology.

## 2. Technological Evolution: From Cytogenetics to Cytogenomics Analysis

**Figure 1:**
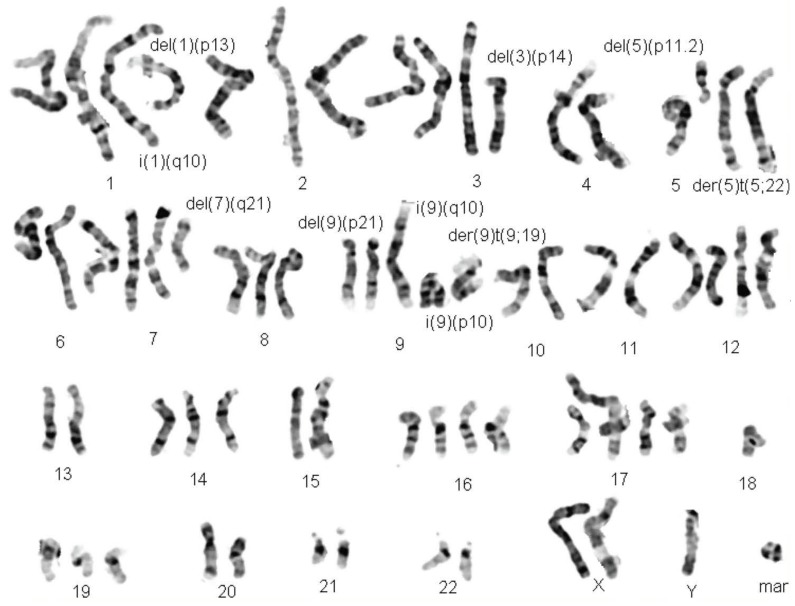
Karyogram of a representative G-banded Head and Neck Cancer metaphase.

**Figure 2:**
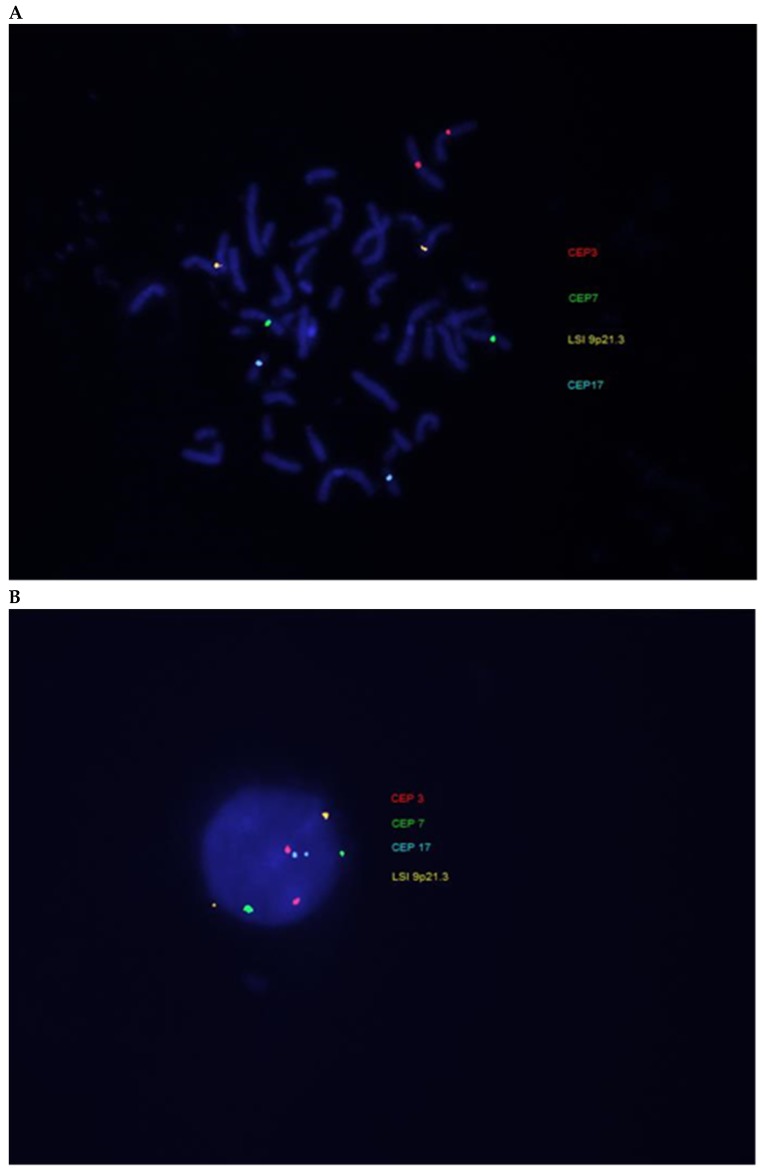
Example of fluorescence in situ hybridization (FISH) technique using UroVysion Bladder Cancer Kit in (**A**) metaphase and (**B**) interphase nuclei.

**Figure 3:**
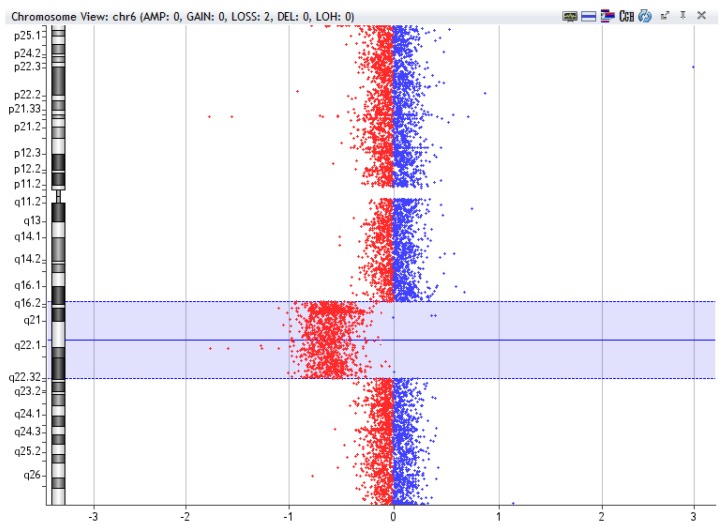
Example of 6q deletion [del(6q16.2-q22.32)], 27,3Mb, detected by array-CGH technique in a sample of Chronic Lymphocytic Leukemia using CytoGenomics (v2.9.2.4) software (Agilent Technologies).

A combination of cytogenetics and molecular biology techniques opened several opportunities in the oncologic field as well as in the precision medicine for oncologic patients. Nowadays, a mixture of low-throughput and high-throughput technologies is used in clinical genetics laboratories allowing the identification of deregulated cellular pathways, mutation signatures, noncoding RNAs and protein expression profiles, with impact in the early diagnosis and prediction of therapeutic response in human cancers. Cytogenetic technique comprises the evaluation of structural and numerical chromosomal aberrations, being Philadelphia chromosome the first chromosomal abnormality discovered in cancer using this technique in 1960 [10], representing the leverage of cancer cytogenetics. In 1970, conventional cytogenetics (also named karyotyping) experienced a revolution with the development of chromosomal banding methods that enabled the identification of individual chromosomes as unique entities based on their specific banding patterns [11] (Figure 1). In 1980, the introduction of molecular cytogenetics was a substantial addition to conventional cytogenetics, since it led to a more precise identification of clinically relevant genetic abnormalities through the observation of specific DNA sequences in the chromosomes and nuclei of cancer cells [12]. The primary technique used for molecular cytogenetic characterization of chromosomes was fluorescence in situ hybridization (FISH) that consists in the hybridization of one or more fluorescently labeled DNA probes to metaphase chromosomes or interphase nuclei [13]. An important advantage of FISH technique to clinical practice was the capability to analyze chromosomal alterations in nondividing cells, namely directly in cytological preparations and tissue sections. Some variants of FISH, such as multiplex FISH (M-FISH), spectral karyotyping (SKY) and comparative genomic hybridization (CGH) brought an easier way to interpret the complex and instable cancer karyotypes, being used in research and clinical cancer detection [12]. FISH techniques improved diagnosis and research field of hematological malignancies and solid tumors [14], providing additional relevant information to that provided by G-banded karyotype. Nowadays, FISH technology is commonly accepted and used in clinical routine setting, due to significant progress that led to (i) increased amount of DNA probes and probe combination availability and (ii) improvement in probe labeling techniques and in optical microscopy [15]. The FISH technique has been widely used in the diagnosis of breast cancer through *HER2* gene amplification detection, of leukemias through *BCR-ABL* gene rearrangements and of non-small-cell lung carcinoma (NSCLC) through *ALK-EML4* gene rearrangements [12]. Furthermore, FISH cancer diagnostic panels have also been used in clinical, namely the UroVysion test (Abbott, USA) for bladder carcinoma screening [16] (Figure 2). In 1992, a genome-wide screening analysis using FISH technology was developed, the comparative genomic hybridization (CGH) technique. In CGH technique DNA from sample in study and the control DNA extracted from an individual with a normal karyotype (46,XX or 46,XY) are differentially labelled with green and red fluorochromes respectively, and equal amounts are co-hybridized in a reference human metaphase, being the difference in DNA content between the control and the DNA in study displayed by a difference in the fluorescence ratios [14,17]. This technique does not require a previous knowledge of the chromosome alterations to be analyzed, since it is not a targeted technique to a specific probe or probe panel but requires good metaphase spreads with long chromosomes. The subsequent development of DNA microarray technologies provided a whole-genome analysis with high-resolution, opening the field of cancer cytogenomics. Indeed, the introduction of DNA probes immobilized in an array format, replacing the conventional metaphase chromosome spreads, enabled the analysis of chromosomal regions with unprecedented detail, revolutionizing the understanding of tumor genomes [18,19] and opening a lot of possibilities for clinical practice (Figure 3). The genome-wide array-CGH technique was initially introduced using cDNA microarrays for gene expression profiling [20]. Nowadays, these genomic and gene expression profiling technologies are used to simultaneously analyze thousands of loci, offering several advantages in a clinical setting [21]. Conventional and molecular cytogenetic analysis identify recurrent chromosomal abnormalities and clonal evolution at cellular level, while cytogenomics technologies such as array-CGH, single-nucleotide polymorphism (SNP) array, and NGS identify at the whole genome level the specific genomic coordinates and cancer-related genes with copy number alterations and mutations [22]. Therefore, during the early 21st century, the progress made in Multiplex Ligation-dependent Probe Amplification (MLPA), array-CGH and NGS technologies, marked another significant step forward in the cancer field and consequently in personalized cancer genomics, revealing specific genomic cancer signatures that allow to classify and group tumors into different subtypes with different behavior and prognosis [23]. MLPA is a multiplex polymerase chain reaction-based method that can detect copy number alterations and point mutations simultaneously, presenting a short hands-on working time [24]. This is a directed technique for a specific set of MLPA probes; almost 50 different genomic locations can be tested in a single reaction, which recognizes target sequences of only 50 to 100 nucleotides in length, being a reliable and robust method to detect specific genetic alterations with diagnostic and prognostic significance [24,25,26]. NGS, also called massive parallel sequencing, is used to sequence a large number of individual genomes in a fast and accurate way; nevertheless, the clinically usefulness of oncological genomic data is difficult to select among the millions of DNA fragments with genetic variations identified in tumors [27]. Nevertheless, due to important improvements in the reliability, sequencing chemistry, pipeline analysis, bioinformatic algorithms, data interpretation, and costs, this technology is now compatible with clinical oncology practice [28]. Besides whole genome or exome analysis, it is also possible to use gene-panels to screen for genes associated with specific cancer types and mutations with therapeutic implications, which combined clinical applicability, cost effectiveness, and the ability to easily identify and interpret relevant genomic alterations [28]. There are different NGS platforms and approaches as well as different possibilities of initial input material to be used, such as genomic DNA (DNA-seq) and messenger or non-coding RNA (RNA-seq) [28]. This approach is important to the identification of gene fusions in solid tumors, since it has been difficult to ascertain due to the limitations of cytogenetic techniques and also the clonal heterogeneity. RNA-Seq overcame these limitations, helping in the discovery of novel recurrent tumor gene fusions arising from chromosomal rearrangements that are not seen at the genomic level and also recurrent chimeric read-through transcripts (as SLC45A3-ELK4 and CDK2-RAB5B) in the absence of DNA alterations [27]. Large consortia and networks as well as clinical trials are using NGS technology to decipher the mutation landscape in several cancer types, proving its clinical utility, namely, the diagnostic and prognostic potential and the response to the individualized cancer treatments [28]. The paradigm has been changing in cancer characterization and patient management with the recent advances in single-cell technologies and with the use of cytogenomic-based integrated analysis, revealing tumor heterogeneity, clonal evolution, and cellular architecture [29]. Single-cell DNA and RNA sequencing methods have several translational applications, such as diagnostics, prognostics, targeted therapy, early detection, and noninvasive monitoring [30]. Besides their capability to detect rare cancer cells, intratumor heterogeneity and molecular alterations, there are some challenges to overcome before the introduction of this technology into routine clinical practice, namely, the huge experimental time and costs as well as the interpretation of results [31]. Moreover, since the first step in any single-cell sequencing experiment comprises whole-genome amplification or whole-transcriptome amplification to get enough input material to build the NGS libraries, there are also several technical challenges associated with the amplification process to be overcome, such as allelic dropout events (one allele is not amplified), amplification distortion (transcripts are over/under amplified), false-positive errors (infidelity of the polymerase), and coverage nonuniformity (irregular amplification) [30,32]. Single-cell RNA sequencing has been the most extensively used approach, improving the knowledge of intratumor heterogeneity, clonal evolution, metastatic dissemination in tumors, and immune landscape of cancer patients [33]. Using this high-throughput technology, the detection and genomic characterization of circulating tumor cells (CTCs) and cell-free DNA emerged recently, opening new possibilities for early tumor diagnosis and the noninvasive monitoring of the disease’s progression/remission using biofluids and exosomes [9].

## 3. Cytogenetic and Genomic Rearrangements in Cancer

The complex process of transformation of a nonmalignant to a malignant cell is described through the sequential acquisition of alterations that lead to sustain proliferative signaling, evade growth suppressors, resist cell death, enable replicative immortality, induce angiogenesis, activate invasion and metastasis as well as the reprogramming of energy metabolism, and evade immune destruction [34]. Therefore, cancer is thought to be a consequence of genomic alteration accumulation, such as single-nucleotide variants (SNVs) and copy number variants (CNVs), and structural rearrangements, which encompass deletions, duplications, inversions, insertions, and translocations that could lead to novel fusion genes [35]. These somatic alterations disrupt tumor suppressor genes or activate proto-oncogenes or both, leading gradually to the accumulation of the hallmarks of cancer [34]. Several acquired genomic alterations in cancer are heterogeneous and identified among different tumors [5]. Some specific numerical and structural cytogenetic alterations have been linked with particular tumor types, being reciprocal translocations, insertions, amplifications, and deletions the most frequent structural chromosomal alterations [11]. Chromosome translocations, inversions, and insertions are frequently found in solid tumors, and balanced translocations are common rearrangements of hematological malignancies [36]. In cancers of epithelial origin, partial deletions, duplications, and unbalanced translocations are the most frequent chromosomal alterations found [37]. High frequency of focal deletions affecting larger genes as *FHIT*, *WWOX*, *PTPRD*, *MACROD2*, and *PARK* in primary tumors of diverse cancer cohorts have been identified [38]. Likewise, *TP53*, *RB1*, *EGFR*, and *KRAS* genes are frequently mutated in diverse cancer types [39]. Nevertheless, it is important to stress that some somatic alterations play a vital role in cancer initiation and progression, but others seem to act as passenger alterations, which do not confer any growth advantage to the cancer cells and do not contribute to tumor development [39]. The majority of tumors include more than one driver gene mutation, like breast, colorectal, and prostate cancers that seem to harbor five to seven driver mutations for cancer initiation and progression, whereas hematological tumors have fewer [40]. The cancer genomic alterations can encompass a short segment of DNA or span over many kilobases of DNA [5]. Moreover, the genesis of some cancer types could be in exogenous sources, namely, DNA sequences from viruses like human papilloma virus, Epstein Barr virus, hepatitis B virus, human T lymphotropic virus 1, and human herpes virus 8 [41]. Somatic mutations in the mitochondrial genomes were also linked to human cancer, but this association needs to be clarified [39].

## 4. Heterogeneity in Cancer

Cancer presents intertumoral and intratumoral variation, since tumors comprise subpopulations of different cells either within a primary tumor (intratumor heterogeneity) or between tumors of different tissues, which include differences of the same tumor type within individuals (intertumor heterogeneity) [42]. Intratumor heterogeneity can appear not only as spatial heterogeneity, representing the irregular distribution of genetically different tumor subpopulations across different disease sites or within a single disease site or tumor but also as temporal heterogeneity, describing the dynamic variations in the genetic diversity of an individual tumor over time [43]. Intertumoral heterogeneity is believed to be the result of patient-specific features such as germline genetic variations, differences in somatic mutation profile, and also environmental factors [43]. The diagnostic and management of cancer patients is difficult due to the presence of tumor heterogeneity, which could be originated by subpopulations of distinct cells with nonrecurring mutations and genomic alterations as well as by clonal evolution and positive selective pressure from therapeutics [44,45]. Intratumor heterogeneity seems to be a pivotal feature that leads to therapeutic failure and consequently to a lethal outcome for numerous cancer patients [46]. Interestingly, the most heterogeneous tumors seem to be those that exhibited high mutation rate and copy number alterations, which is common in lung and skin melanoma, bladder, head, and neck and stomach cancer [47]. Tumor heterogeneity, e.g., the co-existence of distinct subpopulations of cells that harbor different genotypes, could be the explanation for different biological behaviors of similar tumors and, consequently, the different clinical outcomes of the patients. Considering cancer as a heterogeneous disease, effective cancer treatments should address patient-specific cytogenomics alterations and features of the tumor microenvironment [48]. However, intratumor heterogeneity is difficult to measure since only a limited portion of the tumor is usually accessible for molecular evaluation, giving a single and static snapshot of a dynamic disease that continues to evolve [47]. The tumor heterogeneity concept was born when some pathologists observed the morphological heterogeneity of tumor cells using the first compound microscopes; nevertheless, the proof of the existence of subclones with different genetic alterations within the same tumor arose with the development by cytogeneticists of the chromosome G-banding, SKY, and FISH techniques [49]. With the advent of new technologies, such as deep sequencing techniques, the presence and extent of both intra and inter-tumor heterogeneity have been described in several cancer types, namely glioblastoma [50], non-small cell lung cancer (NSCLC) [51], renal cancer [52], breast cancer [53], prostate cancer [54], and ovarian cancer [44]. There is a growing knowledge related to genetic diversity within and between several tumors, but how such diversity is generated and its implications upon clinical outcomes, namely, the response and the resistance to therapies, are still unclear [55]. Tumor heterogeneity may also hinder the identification of molecular predictive biomarkers, hampering their successful translation to clinical practice with benefit to patients. Recent single-cell sequencing technology allows the isolation and characterization, with high resolution, of individual cells within a mixed population, which encloses great potential to shed light into the multiple layers of intratumor heterogeneity [43]. Integrative cytogenomic multiplatform analysis of single cancer cells seems to be pivotal for a more comprehensive molecular classification of tumors, which will have great usefulness and impact for cancer patient management and clinical decision making [45].

## 5. Biomarkers in Cancer

The gold standard in cancer control and prevention is the early detection as asymptomatic malignancies or in some instances even as potentially malignant lesions [56]. This could be achieved through the identification of biomarkers that will be specific cancer-associated alterations. These will certainly be able to assist in cancer diagnosis and prognosis, stratify patient risk, predict sensitivity or resistance to a specific therapy, and monitor treatment response or residual disease. Identifying the molecular profile of each cancer type is vital to perform the genotype–phenotype correlation and consequently to establish biomarkers with clinical applications [57]. The recent revolution of the high-throughput technologies allows a deep cytogenomic characterization of tumor tissue, opening several challenges in the clinical translation of these molecular big data [58]. Presently, there is substantial progress in the identification and validation of novel cancer biomarkers. We are witnessing a change from a ‘one-size-fits-all’ concept to a precision medicine reality, which has proven to be a very complex task [59]. Therefore, there are some molecular biomarkers in clinical practice routine and others are under investigation or under clinical trial evaluation [58,60]. Association of DNA copy-number alterations with prognosis has been found for numerous tumor types, namely prostate cancer [61], breast cancer [62], gastric cancer [63], and lymphoma [64]. In spite of numerous prognostic cancer biomarkers being reported in the literature, only few biomarkers have been approved for clinical practice that could change clinical decision making, helping in the therapeutic choices and patient management [58], showing the complexity of cancer and the lack of a strong bridge between the laboratory and clinicians. Genomic biomarker implementation in clinical practice has proven to be tremendously hard despite progress with the human genome sequence and the development of high-throughput technologies [59]. Overexpression/amplification of *HER2* (*ERBB2*) is used to predict the response to monoclonal antibodies such as trastuzumab and pertuzumab in breast cancer [65] and the response to trastuzumab in esophago-gastric adenocarcinoma [66]. Examples of other predictive biomarkers with clinical relevance to targeted therapies are the BCR-ABL tyrosine kinase, a fusion gene that is associated with Philadelphia chromosome in chronic myeloid leukemia [67], KRAS mutations in colorectal cancer [68], and a mutation in the *KIT* proto-oncogene in the gastrointestinal stromal tumor [69]. Mutations in *EGFR*, *KRAS* and driver mutations in *ALK*, *BRAF*, *PIK3CA*, *AKT1*, *MAP2K1*, and *MET* in NSCLC [70] are also examples of predictive biomarkers as well as *BRAF* mutation in melanoma [71]. Significative progress in gene expression evaluation with improved understanding of the roles of altered genes in malignant transformation allowed to customize the diagnostic and therapeutic tools [72]. In breast cancer, there are several gene expression signatures that can be used to estimate prognosis for an individual patient based on assessment of the tumor [73], *mammaprint* being one of the first gene expression signature-based assays to predict breast cancer recurrence after chemotherapy [74]. Likewise, Oncotype DX genomic prostate score is based on quantification of gene expression, allowing to assess cancer aggressiveness, recurrence after radical prostatectomy, and metastases [72]. Despite precision cancer medicine having been recognized as a research priority and vital strategy to improve oncological outcomes, the progress in the clinic has been slow [59]. The search for cancer biomarkers is an ongoing work that needs standardization of genomic analysis to improve accuracy and consistency of the new findings [75]. A paradigm shift is emerging from analysis of targeted sequencing panels of specific selected point mutations to genome-wide assays at different omics levels, which requires important developments in computational biology to data analysis and interpretation with phenotype correlations. With the advent of new high-throughput technologies, novel targeted therapies continue to be developed, which underscored the needed of translate to cancer patient clinic routine management new omics biomarkers [75]. Nowadays, array-based methods, deep sequence technologies, and integrative genomic analysis help in the identification of robust and reliable genomic tools to guide personalized treatment decisions and to improve oncological outcomes [59]. Currently, the use of body fluids, also called liquid biopsy, for the quantification and specific biomarkers identification in circulating cells and circulating DNA and RNA has attracted great interest, allowing a non-invasive or minimally invasive way to early diagnose tumors, to monitor real-time dynamics of cancer and patient follow up [9,76]. Liquid Biopsies enclose a great promise since tissue biopsies often do not reflect intratumor genetic heterogeneity and tumor behavior, with sequential repetition of multi-site biopsies being unpractical. However, their clinical application has been hampered by several technical challenges and by the lack of standardization of preanalytical and analytical variables [77]. In 2016, the first ctDNA-based diagnostic test (cobas1 EGFR Mutation Test v2; Roche Diagnostics) was approved by the U.S. Food and Drug Administration (FDA), used to guide the application of epidermal growth factor receptor (EGFR)-tyrosine kinase inhibitors on the basis of specific EGFR-sensitizing mutations in patients with NSCLC [77,78]. Moreover, the test for enumeration of epithelial CTCs (CellSearch® platform) is the only FDA-approved clinical application of CTCs, used for metastatic breast, prostate and colorectal cancer [79].

## 6. Conclusions and Future Perspectives

Progress in high-throughput technologies has been enabling a better understanding of molecular alterations, biological mechanisms, and behavior of different human cancer types. The cytogenetics and cytogenomics advances together with bioinformatics have the potential to identify specific molecular signatures linked to tumor initiation and progression as well as to effective therapeutic targets. Therefore, the current improvement of genome-wide techniques revolutionized the oncologic diagnostic field, through gene expression profiling, array-CGH, single-nucleotide polymorphism array, and NGS that allow the analysis of a specific gene or of all genome or exome as well as gene interactions and pathways that are altered in cancer. The promise of big data generated from multiple high-throughput technologies to identify and establish biomarkers for early cancer diagnosis, for prognosis, and for therapeutic response does not have yet a significant translation to clinical practice because there are numerous candidate biomarkers for several cancer types, and only few are ready for clinical use. Looking ahead, significant improvements in cancer diagnosis and treatment during the coming decades seems to be a truthful belief, achieved by triggering a better and more comprehensive understanding of tumor biology and heterogeneity, with identification of multiple molecular alterations that drive cancer development ultimately guiding personalized cancer treatments. Human cancer research will still benefit from improvements in high-throughput technologies, helping in the identification of targetable mutations and gene fusions. Moreover, nowadays, these technologies allow to profile the molecular landscape of tumors with time and costs almost compatible with diagnostic setting. These high-throughput technologies present great potential to pursue the precision medicine applications, but some challenges regarding data interpretation and their real practical applicability to patients benefit need to be overcome. Some clinical applications of the big data generated by these high-throughput technologies aim to improve cancer patients outcomes as well as to identify new targeted therapies and consequently select the patients that will benefit from those therapies based in the tumor specific genomic signatures, being vital multi-omics integrative and validation studies. In this sense, it is expected that decreased cost of high-throughput technologies will empower clinicians to tailor therapeutic strategies according to the molecular profile of each tumor. Nevertheless, it is important to stress that usually a single molecular analysis of a small portion of tumor tissue is only performed, which does not reflect the whole picture of tumor biology. Single cell sequencing opens new possibilities to reveal tumor heterogeneity as well as therapeutic resistance and sensitivity; however, in a routine clinical practice this technology is still unreachable, needing to overcome some methodological and data interpretation limitations. Multidisciplinary teams with collaborations between clinicians, clinical laboratory geneticists, researchers, and bioinformaticians are pivotal to identify, interpret, and validate the molecular data and consequently to translate these data with success to clinical practice.
